# Precision medicine for gastrointestinal cancers: a conference report

**DOI:** 10.2144/fsoa-2020-0061

**Published:** 2020-05-27

**Authors:** Ibrahim Halil Sahin

**Affiliations:** 1 Emory University School of Medicine, Winship Cancer Institute, Atlanta, Georgia, USA

**Keywords:** cholangiocarcinoma, colorectal cancer, gastric cancer, gastrointestinal cancers, hepatocellular carcinoma, IDH mutations, immunotherapy, pancreatic cancer, precision medicine

## Abstract

As cancer management evolves into precision medicine national/international cancer meetings bring novel therapeutic approaches and potentially practice-changing results of clinical studies are presented. This year, the ASCO GI Symposium 2020 had also several updates from ongoing and finalized clinical trials. Although there were no groundbreaking results that impact the daily practice directly, several highly important data from ongoing studies were shared with the audience. In this report, the highlights of the ASCO GI Symposium 2020 are presented with a future perspective.

The management of gastrointestinal cancers has substantially evolved into personalized medicine in the last decade, due to a better understanding of molecular underpinnings of each and unique gastrointestinal cancer [1]. While the oncology society has improved in learning how to provide the best care for an individual cancer patient, the novel coronavirus, SARS-CoV-2, has caught oncologists around to the world of the guard with an extremely challenging situation where finding the balance between the risk of infection due to chemotherapy and even due to hospital visits versus the risk due to acuity of controlling cancer progression. While the ASCO GI Symposium 2020 has focused mostly on outcomes of recent ‘high-yield’ clinical trials, perhaps one of the most important discussion points of the ASCO GI Symposium 2021 would be risk-adapted cancer management in pandemics. The impact of ongoing COVID-19 infection on the management of cancer patients will be better characterized once the acute phase of this pandemic is over.

The first day of the ASCO GI 2020 Symposium had mostly presentations and discussions on upper gastrointestinal (GI) cancers. The FLOT4 trial [2], which showed superior overall survival (OS) outcomes with the use of perioperative fluorouracil, leucovorin, oxaliplatin (known as FLOT regimen) as compared with perioperative epirubicin, cisplatin with fluorouracil or capecitabine (median OS: 50 vs 35 months, hazard ratio [HR] of 0.77; [CI: 0.63–0.94]) has brought a new debate to the oncology field for the management of locally advance resectable gastroesophageal junction (GEJ) adenocarcinoma. After this study, the practice has at least partially shifted away from preoperative chemoradiation (CRT) given significant improvement in outcomes without the requirement of radiation therapy [3]. At this time, there is no available data comparing FLOT to CRT. Although there is no consensus given lack of data from randomized trials, patients with a higher risk of systemic recurrence such as patients with high local lymph node tumor burden (such as N2 disease) should be considered for perioperative FLOT4, while patients with large local tumors with no local lymph nodes can be managed with preoperative CRT. The ESOPEC trial is currently comparing the FLOT regimen with CRT and the results of this study will answer this question (NCT02509286). At this juncture, positron emission tomography-computed tomography (PET CT) can be utilized as an important tool for response assessment in GEJ adenocarcinoma patients who are undergoing FLOT or CRT to personalize the treatment based on the initial response and adjust the treatment if necessary. Blood-based molecular markers (such as specific mutation determined in primary tumor) for surveillance during the post-surgical period, for minimal residual disease, continue to evolve and likely will be a part of the standard of care in oncology practice, including GEJ adenocarcinoma patients.

The management of advanced-stage GEJ/gastric adenocarcinoma patients has been embracing significant changes in the last couple of years. Although no practice-changing data was presented in the ASCO GI Symposium 2020, the management of advanced-stage GEJ/gastric adenocarcinoma patients is evolving into highly molecular-based approach, with initial molecular characterization to determine important therapeutic markers such as mismatch repair deficiency, PD-L1 expression, and HER2 status at the time of diagnosis is recommended. Pembrolizumab, an immune checkpoint inhibitor targeting PD-1, has shown promising activity, particularly in the first-line setting as compared with the subsequent line of therapy (KEYNOTE 59 and KEYNOTE 60) [4–6]. The benefit appears to be limited only to a subgroup of GEJ/Gastric cancer patients with positive PD-L1 expression (KEYNOTE-062) [7]. Notably, the benefit was significant for OS but progression-free survival (PFS) did not improve with the use of pembrolizumab as compared with chemotherapy. Currently, the effectiveness of pembrolizumab is also being investigated in GEJ/gastric cancer patients with *HER2 amplification* (KEYNOTE 811) [8]. Overall, pembrolizumab can be considered either in the first or second line of therapy in patients with high expression PD-L1 (combined positive score (CPS) ≥10%), albeit more molecular markers are needed in this field to better identify right patient population who may benefit from these agents. At last, this agent should be strongly considered in the first-line setting for MMR-D GEJ/gastric cancer patients given that highly durable clinical response has been well established in the field.

The targetability of the pancreatic cancer microenvironment has been investigated in multiple studies. Multiple agents that targeting dense stroma at multiple levels have failed to demonstrate any clinical activity including sonic hedgehog inhibitors and stroma modifying agents [9]. One the disappointing, yet not surprising, findings reported at the ASCO GI Symposium 2020 was the lack of efficacy of another stroma modifying agent, PEGPH20, which is pegylated recombinant human hyaluronidase. This novel agent was combined with gemcitabine and nab-paclitaxel in metastatic pancreatic adenocarcinoma patients and did not improve survival outcomes (HALO-3 trial) [10]. PEGPH20 was previously combined with the FOLIFINOX regimen in Phase Ib/II trial and that study demonstrated the detrimental outcomes in the investigational arm with the use of this agent [11]. At this time, the stroma depletion approaches with hyaluronidase do not offer any hope for the future. The phase III trial of pegylated IL-10, which aimed to optimize the tumor microenvironment of pancreatic adenocarcinoma to improve antitumor immunity, did not add any benefit when combined with FOLFOX as a second line therapy [12]. Collectively, this growing evidence indicates that pancreatic cancer microenvironment carries very complex dynamics and simply depleting the tumor stroma will likely continue to fail to demonstrate any significant clinical efficacy. It is perhaps an ideal time to go back to the bench to better understand the microenvironment and cancer cell interaction in the pancreatic adenocarcinoma field.

The progress in homologous recombination deficient pancreatic cancer continues to evolve. The addition of veliparib to cisplatin and gemcitabine did not result in improvement in PFS or OS. However, cisplatin and gemcitabine combination arm achieved an objective response rate of 65.2% among pancreatic cancer patients with homologous recombination deficient (*BRCA1/2* and *PALB2* mutations), which makes it an alternative to FOLFIRINOX in this subset of patients [13].

The efficacy of immune checkpoint inhibitors in hepatocellular carcinoma (HCC) continues to evolve with different combinations. Recently, the IMPOWER 150 trial changed the standard of care of advanced-stage hepatocellular carcinoma to the combination of atezolizumab and bevacizumab as a first-line treatment [14]. The combination of nivolumab and cabozantinib with or without ipilimumab is now being investigated in HCC patients with or without previous sorafenib exposure and preliminary outcomes from phase I/II study demonstrating overall promising results (objective response rate of 17% with doublet and 26% with triplet) [15]. Overall this trajectory suggests that we may see further therapeutic strategies with the combination of tyrosine kinase inhibitors and immune checkpoint inhibitors for the treatment of HCC. Cholangiocarcinoma is another heavily studied field for targeted therapies and it is highly enriched with potentially actionable genes. For example, *IDH 1/2* mutations are currently actionable mutations with US FDA-approved drugs and are highly detected in biliary tract cancers with most notably among intrahepatic cholangiocarcinoma (approximately 17% of patients) among all biliary cancers [16]. Currently, clinical trials are investigating IDH1/2 inhibitors in biliary cancer patients with *IDH* mutations [17]. Multiple other exciting targeted therapies are also being rapidly developed for biliary tract cancer patients that include agents targeting fibroblast growth factor receptor alterations, *HER2 amplification*, *BRAF* *V600* mutations and HRD pathway [18], which will move us one more step to precision medicine.

The efficacy of immune checkpoint inhibitors in mismatch repair deficient (MMR-D) colorectal cancer has been well established as a subsequent line of therapy. Currently, clinical trials investigating these agents in the first-line setting with promising clinical outcomes [19]. Most of the clinicians have already implemented these agents as first-line therapy given highly durable responses and more to come in this field. Notably, a multicenter study investigating the biomarkers of immune checkpoint inhibitor response in patients with MMR-D colorectal cancer identified *BRAF V600* mutations and specific types of MMR genes as a predictor of worse 12- and 24-month PFS rates [20]. Future prospective studies are needed to better understand the predictive value of *BRAF* *V600* mutations in MMR-D colorectal cancer patients who are treated with immune checkpoint inhibitors. This novel finding may trigger novel therapeutic approaches for BRAF-mutant MMR-D colorectal cancer patients.

BRAF box is now open and *BRAF V600* mutations are now among targetable genomic alterations in metastatic colorectal cancer patients. The combination of BRAF ± MEK inhibitors with anti-EGFR monoclonal antibodies has achieved promising responses in this highly aggressive subset of colorectal cancer patients leading to improvement in survival and quality of life outcomes [21]. Locoregional treatment approaches for oligometastatic colorectal cancer, which often aims a curative intent continues to prove effective and improve survival outcomes [22]. Proceeding aggressive systemic disease control with triplet chemotherapy before locoregional approached appears to have a positive impact on oligometastatic colorectal cancer patients [21]. On the other side of the equation, surgical resection of the primary tumor in incurable metastatic colorectal cancer patients does not add any benefit [23] suggesting that aggressive surgical approaches should be avoided for incurable colorectal cancer patients, unless otherwise indicated. A comprehensive analysis from the national database presented in ASCO GI Symposium 2020 reported worse survival outcomes in young adults with colorectal cancer and social disparities confirming the growing concern regarding the inability of young adults to access healthcare [24]. These alarming results indicate the existence of a significant gap in the healthcare system of the USA where young adults have difficulty accessing the medical care even for cancer care which needs to be addressed in the near future.

Another growing scientific discussion in cancer research embraces the type of biopsy utilized for molecular profiling. Currently, the gold standard of molecular profiling is tissue biopsy. However, at the time of disease progression, performing liquid biopsy-based circulating tumor DNA analysis may provide further information perhaps particularly on disease heterogeneity, as well as novel mutations evolved through the progression of disease that may be actionable. Circulating tumor DNA can be also considered where tissue biopsy is not available or accessible in current clinical practice. Liquid biopsies are often being used in clinical trial protocols where specific molecular alterations are tested during the initial evaluation of patients for biomarker-driven trials. More research is indicated in that field to better characterize the use of liquid-based molecular profiling perhaps, more importantly, their sensitivity to specific molecular alterations such as HER2 amplification and microsatellite instability status.

Collectively, the advances in the management of gastrointestinal cancers are highly promising for the future. The cancer management is evolving into personalized medicine than ever before as we better understand disease heterogeneity led by distinct molecular alterations for individual tumor type. Although this progress may bring some challenges such as financial toxicity and requirement more collaborative work to identify patients with specific molecular alterations to successfully conduct biomarker-driven clinical trials, precision medicine provides significant hope for cancer patients for improved quality and length of life.
